# Oral Hygiene Status in Rheumatoid Arthritis Patients and Related Factors

**DOI:** 10.31138/mjr.32.3.249

**Published:** 2021-09-30

**Authors:** Saloua Afilal, Hanan Rkain, Afaf Allaoui, Safaa Fellous, Jihad Moulay Berkchi, Fatima Zahrae Taik, Ilham Aachari, Latifa Tahiri, Nada Alami, OumKeltoum Ennibi, Najia Hajjaj-Hassouni, Fadoua Allali

**Affiliations:** 1 Rheumatology B Department, El Ayachi Hospital, Faculty of Medicine and Pharmacy of Rabat, Mohammed V University, Rabat, Morocco; 2 Laboratory of Physiology, Physiology team of Exercise and Autonomic Nervous System, Faculty of Medicine and Pharmacy of Rabat, Mohammed V University, Rabat, Morocco; 3 Periodontology Department, Faculty of Dentistry, Mohammed V University, Rabat, Morocco; 4 International University of Rabat, Morocco

**Keywords:** rheumatoid arthritis, joint disease, oral hygiene, brushing

## Abstract

**Objectives:**

To evaluate oral hygiene status in Rheumatoid arthritis (RA) patients, to analyse possible related factors, and to investigate the role of the rheumatologist in information about importance of adequate oral hygiene status in RA patients.

**Methods:**

A cross-sectional study that included 100 consecutive RA patients (89% female, mean age 46.7 ± 11.7 years). For each patient, we recorded oral symptoms, oral hygiene status and role of rheumatologist in information on the oral hygiene status. Factors associated with regular brushing (≥2/day) were also analysed.

**Results:**

Median disease duration was 8 years (4;2). Dental pain was reported by 74% of patients and bleeding by 51% of them. Regular brushing was noted in 45% of patients. The use of a correct brushing method was noted in 14% of cases. Two patients reported visiting a dentist regularly. Information explaining that poor oral hygiene has a negative impact on RA was delivered by rheumatologist to 11 patients. Regular brushing of teeth was recommended by rheumatologist to 8 patients and 10 patients were advised by their rheumatologist to consult a dentist. Regular brushing was more important in women (48,3% vs 18,2%; p=0.05) and in the literate patients (57,6 vs 31,2%, p<0.01). No association was found between regular brushing, Disease Activity Score 28 (DAS28) and health Assessment Questionnaire (HAQ).

**Conclusion:**

This study illustrates bad oral hygiene status in RA patients, which seems more important in men and illiterate patients. It also highlights poor information given by the rheumatologist.

## INTRODUCTION

The World Health Organization (WHO) defines oral health as a state of being free from mouth and facial pain, oral and throat cancer, oral infection and sores, periodontal disease, tooth decay, tooth loss, and other diseases and disorders that limit an individual’s capacity in biting, chewing, smiling, speaking and psychosocial wellbeing.^1^ Good oral hygiene helps fight against periodontal diseases due to the presence of bacteria that accumulate in the mouth as a complex biofilm.

Rheumatoid arthritis (RA) is the most common chronic inflammatory rheumatism. It affects women about four to five times more often than men under 50 years of age, but this difference seems to diminish with age.^2,3^ In addition to alterations in systemic immune function, RA causes an accumulation of proinflammatory cell infiltrates in the synovial membrane, which leads to synovitis, destruction of cartilage and bone tissue of the joints, and, finally, to physical impairment and disabilities.^3,4^ All joints can be affected by RA, but the wrist, proximal interphalangeal joints, and metacarpophalangeal joints are the most frequent and early affected,^3^ which may lead to important manual disability. Oral hygiene can be impaired in these patients, making them predisposed to dental plaque accumulation and, therefore, inflammatory periodontal disease.^5^

Several studies have shown the link between RA and periodontal disease.^5–13^ This epidemiological association has been described for several decades.^14^ These two diseases are remarkably similar in their physio-pathological mechanism, which involves common cellular and molecular mechanisms.

The primary objective of this study is to evaluate oral hygiene status in a Moroccan population of patients with RA. We also aimed to investigate the role of the rheumatologist in information about importance of adequate oral hygiene status in RA patients and to determine the factors possibly associated with the precariousness of measures of oral hygiene.

## MATERIALS AND METHODS

This is a cross-sectional study in collaboration with the Periodontology department of Faculty of Dentistry Rabat Morocco. The study was approved by the ethics committee of the University Mohammed V Rabat (Faculty of Medicine and Pharmacy) and was conducted in conformity with ethical principles of research. A total of 100 subjects were consecutively included in the present study. Subjects with RA were recruited from individuals who attended the Department of Rheumatology El Ayachi hospital (public structure and referral hospital of Rheumatology in Morocco, where hospitalisations and outpatient clinics are accessible for patients originally from different regions of our country) for routine examination or for hospitalisation. RA was diagnosed according to the criteria of American College of Rheumatology ACR / European League Against Rheumatism EULAR 2010.^15^ RA patients that were totally toothless were excluded from this study. Between February and June 2018, 100 subjects with RA provided informed consent and were enrolled into the study.

### Data collection

Patients completed a questionnaire. The questions were closed-ended, single-choice, or multiple-choice. Because many patients were illiterate, the questionnaire was administered and completed by a physician.

The questionnaire comprised four sections, of which the first collected demographic and socioeconomic data: patient’s age and gender, whether residing in an urban (a vast, orderly, furnished space, with pleasant architecture and including all the services necessary for a lifestyle) or rural community (a small space dominated by agricultural activity alone and which is in a situation of lack and disqualification due on the one hand to the predominance of this agricultural activity, and on the other hand, due to the lack of services that the city offers), formal education (none, primary school, secondary school, university education), hospitalised or outpatient, comorbidities (diabetes [defined as random blood glucose RBG >11.1 mmol/L, or fasting blood glucose FBG ≥7 mmol/L, or being on diabetes medication], arterial hypertension [defined as systolic blood pressure ≥140 mmHg and/or diastolic blood pressure ≥90 mmHg, or currently taking medication for hypertension], dyslipidaemia [defined as abnormal TC, LDL-C, HDL-C, and/or triglyceride or being on dyslipidaemia medication], dysthyroidism [hypothyroidism or hyperthyroidism or already under treatment for dysthyroidism]), smoking (active or passive), a n d menopause status for women.

The second section of the questionnaire supplied information on the joint disease: age at onset, delay in referral for specialist care, disease duration, disease activity evaluated by Disease Activity Score 28 (DAS28),^16^ functional status assessed by score on the validated Arabic version of the Health Assessment Questionnaire (HAQ),^17^ therapeutics used at the time of the study (corticosteroids, conventional synthetic disease-modifying anti-rheumatic drugs [csDMARDs], biologic DMARDs). The third section gathered data on the oral hygiene status at the time of study: oral symptoms (dental pain, bleeding, bad breath), brushing teeth (daily brushing frequency, brushing time [<3 or ≥3 min], brushing method), brushing equipment (type of toothbrush, type of toothpaste, frequency of change of toothbrush), regular dentist visit (yes/no), and patient’s perception of his oral health status (very good, satisfying, bad, or very bad). The last section focused on the place of the rheumatologist in information about importance of adequate oral hygiene status in RA patients. To investigate this topic, 4 questions were asked of patients: if their rheumatologist has ever examined their oral cavity, if they have ever been informed that poor oral hygiene has a negative impact on their RA, if their rheumatologist ever recommended regular brushing of their teeth, and if they recommended to them to consult a dentist.

### Statistics

Quantitative variables were expressed in means and standard deviation or in medians and quartiles, depending on whether their distribution is symmetrical or not. The qualitative variables were expressed in frequencies. Statistical analysis data was performed to identify possible factors associated with precarious oral status. Depending on the nature of the associations sought, various statistical tests were used: Student’s t-test, Mann-Whitney and chi-squared test. P values less than 0.05 were considered significant. The software used to process the data collected was the SPSS (Statistical Package for Social Science), SPSS Inc., Chicago, Version 21.0.

## RESULTS

### Patient characteristics

100 RA patients were included (11 males and 89 females, mean age of 46,7 years ± 11,7, median disease duration of 8 years (4;2). Place of residence was rural in 10% of patients and urban in 90%. Illiteracy rate was 48%. Diabetes concerned 9% of patients, arterial hypertension 10%, dyslipidaemia 7%, and dysthyroidism 8%. Active smoking was noted in 3% of patients and menopause concerned 50.6% of women. (**Table 1**)

**Table 1.** Characteristics of RA patients.

**Characteristic**	**N=100**

**Age** (years)^*^	**46,7 ± 11, 7**

**Female gender** (%)	**89**

**Place of residence: Urban / Rural (%)**	**90/10**

**Education level:**	
- Illiteracy (%)	**48**
- Primary (%)	**33**
- Secondary (%)	**14**
- University (%)	**5**

**Hospitalization / outpatient** (%)	**61/39**

**Comorbidities:**	
- Diabetes (%)	**9**
- Arterial hypertension (%)	**10**
- Dyslipidaemia (%)	**7**
- Dysthyroidism (%)	**8**

**Smoking:**	
-Active smoking (%)	**3**
-Passive smoking (%)	**11**

**Menopause** (%)	**50,6**

N=patients’ number;

* Mean and standard deviation

### RA characteristics

Mean age at RA onset was 35.8 ± 12.9 years. Median of delay in referral for specialist care was 3 months (0;24) and median of RA duration was 8 years (5;14). Patients have a DAS28 at 4,2 ± 1,8 and median of HAQ score was 1.1 (0.4; 1.9). Among our patients, 81% were under steroids, 57% under methotrexate and 20% under biologic DMARDs. (**Table 2**).

**Table 2.** Characteristics of RA.

**Characteristic**	**N=100**

Age at RA onset (years)	**35,8 ± 12,9**
Referral to specialist delay (Month) ^*^	**3 (0; 24)**
RA duration (Years) ^*^	**8 (5; 14)**
VAS (0–10) ^*^	**4 (2; 6)**
ESR (mm / 1st H) ^*^	**23 (14; 43)**
DAS28	**4,2 ± 1,8**
HAQ ^*^	**1,1 (0,4; 1,9)**
Corticosteroids (%)	**81**
-Daily dose of corticosteroids (mg)	**6,9 ±3,3**
csDMARDS (%):	
- Neither	**21**
- Methotrexate (MTX)	**57**
- Leflunomide	**5**
- Sulfasalazine (SLZ)	**10**
- Hydroxychloroquine (HCQ)	**7**
Biologic DMARDs (%)	**20**

* Values are presented as median and quartiles.

VAS: Visual Analogue Scale; ESR: Erythrocyte Sedimentation Rate; DAS28: Disease Activity Score; HAQ: Health Assessment Questionnaire; csDMARDS: conventional synthetic disease-modifying anti-rheumatic drugs

### Oral hygiene status

Oral symptoms reported by patients were respectively dental pain, bad breath, and bleeding in respectively 74, 51 and 52% of cases. Among our patients, 82% brush their teeth at least once a day and 45% respect the regular frequency of brushing (≥ 2 times/day). Among those who brush (N=82), 31% respect the adequate brushing time (≥ 3min), 67.1% use a commercial toothbrush and 14% use a correct brushing method. (**Table 3A,B**)

Table 3.Oral hygiene status in RA patients.

**Table 3A.** Daily brushing frequency in RA patients (N=100).

**Items**	**N=100**
**Daily brushing frequency (%)**	
- 0 times / day	**18**
- Once a day	**37**
- 2 times / day	**34**
- 3 times / day	**8**
- After each meal	**3**

**Table 3B.** Brushing characteristics in RA patients who brush their teeth (N=82).

**Items**	**N=82**
**Adequate brushing time (≥3min) (%)**	**31**
**Correct brushing method (%)**	**14**
**Type of toothbrush used (%)**	
- Commercial	**67.1**
- Pharmaceutic	**31.7**
- Electric	**1.2**
**Frequency of toothbrush change per year (%)**	
- Every 3 months	**42.6**
- 3-6 months	**35.4**
- > 6 months	**22**
**Type of toothpaste used (%)**	
- Fluoride	**93.9**
- No-fluorinated	**6.1**

Among our patients, 67% perceive that they have bad or very bad oral health status. Regarding dentist visitation, 2 patients who visited regularly the dentist.

### Place of the rheumatologist in information about importance of adequate oral hygiene status in RA patients

Patients reported that their rheumatologist had ever examined their oral cavity in 6% of cases. Information explaining that poor oral hygiene has a negative impact on RA was delivered by rheumatologist to 11% of patients. Regular brushing of teeth was recommended by rheumatologist to 8% of patients and ten patients (10%) were advised by their rheumatologist to consult a dentist. (**Table 4**)

**Table 4.** Place of information on oral hygiene in rheumatologic care.

**Questions**	**Yes (%)**
1. Has your rheumatologist ever examined your oral cavity?	**6**
2. Have you ever been informed by your rheumatologist that poor oral hygiene has a negative impact on your rheumatoid arthritis?	**11**
3. Does your rheumatologist ever recommended regular brushing of your teeth?	**8**
4. Has your rheumatologist already recommended you consult a dentist?	**10**

### Factors associated with regular brushing

Regular brushing (≥ 2/day) was significantly more important in the female sex (48,3% vs 18,2%; p=0.05) and in the literate patients (57,6 vs 31,2%, p<0.01). No association was found between regular brushing, DAS28 or HAQ. (**Table 5**)

**Table 5.** Analysis of factors associated with regular brushing ≥ 2/day.

**Items**	p value^*^	**Brushing ≥ 2/day**		p value^*^
		**YES**	**NO**	
Sex^*^	Female	43	46	0.05^*^
	Male	2	9	
Age^**^				0.4
Illiteracy^*^	Yes	15	33	<0.01^*^
	No	30	22	
Place of residence ^*^	Urban	47	43	0.1
	Rural	2	8	
Menopause^*^	Yes	20	25	0.5
	No	23	21	
RA duration^***^				0.5
Referral to specialist delay ^***^				0.5
VAS^***^				0.9
ESR^***^		45	55	0.18
DAS28^**^		45	55	0.1
HAQ^***^				0.5
Corticosteroids^*^	Yes	40	41	0.08
	No	5	14	
Methotrexate^*^	Yes	27	30	0.6
	No	18	25	
Biologic DMARDs^*^	Yes	8	37	0.6
	No	12	43	

P significant if ≤ 0.05, statistical tests: chi-squared test (*);

Student’s t-test (**);

Mann-Whitney (***).

## DISCUSSION

The present study showed a precarious oral hygiene in a sample of RA patients in Morocco. It showed also an important lack of delivering information by a rheumatologist about importance of adequate oral hygiene status in RA patients. Poor oral hygiene seems to be more prevalent in men and illiterate patients.

Our findings indicate that approximatively one fifth of patients never brush their teeth. Less than half of patients brush their teeth regularly. Only one-third of them respects the adequate brushing time and one fifth used inadequate brushing method. These results illustrate an evident bad status of oral hygiene in our context.

The majority of patients who brush their teeth, use a commercial toothbrush instead of pharmaceutical ones. This can be explained by the higher price of the pharmaceutical toothbrush which is three times that of the commercial toothbrush. We also found that only 1 patient used an electric toothbrush. This is surprising since electric toothbrush seems to be a good alternative for RA patients whose movements necessary to perform brushing can be difficult and painful.

Adequate brushing is also linked to the quality of the toothbrush. However, more than half of patients did not change their toothbrush every 3 months.

Almost all patients used a fluoride toothpaste, since toothpaste in Morocco contain usually fluorine.

Majority of our patients reported dental pain, bad breath or bleeding. Moreover, more than half of them were aware that their oral health status was bad or very bad. In spite of this, only 2 patients visited regularly the dentist. This may be explained by the cultural factors involved in poorer oral health of Moroccan population and an inadequate implementation of preventive strategies regarding their oral health despite their knowledge of them.^18^

The link between RA, oral health, and periodontitis is well known. These two diseases are remarkably similar in their physio-pathological mechanism, which involves common cellular and molecular mechanisms. Improvement of oral hygiene is associated to lower disease RA activity.^19^ Contrasting with this evidence, we are alarmed by our clinical practice data. In fact, our study illustrates the profound lack of delivering information by rheumatologist about importance of good oral hygiene status in RA patients. Only a minority of Moroccan rheumatologists focused on oral hygiene in rheumatologic care of their RA patients. Those results pointed out an urgent need for rheumatologist sensitisation toward more implication in discussing importance of adequate oral hygiene status with RA patients.

Our study has also analysed associated factors to regular brushing of teeth. The high prevalence of regular brushing in women could be explained by women’s interest in the self-image. Our results are comparable with those of a previous study.^20^

Regular brushing was also associated with illiteracy. This finding is in concordance with a study conducted by Pinho MdN et al. Their data showed that the plaque index was more important in illiterate subjects and was linked to a precarious level of oral hygiene.^21^ This result is not surprising since illiteracy is often linked to poor socio-economic status and then to a bad oral hygiene. No relationship was found between regular brushing and disease activity. This could be explained by the heterogeneous nature of the studied population and the cross-sectional nature of our study. To better evaluate this relationship, it would be interesting to complete by a longitudinal follow-up of patients who must be made aware of the importance of oral hygiene.

Functional disabilities of the upper limbs in RA patients may contribute to poor manual dexterity with the toothbrush and lower oral hygiene status.^22^ As reported by previous studies [8,9] plaque accumulation seems to increase in RA patients with functional disability. In our study, no link was found between oral hygiene and functional status assessed by HAQ score. Those results concorded with previous studies.^6,10,23^ Indeed, the HAQ score is a validated functional score in RA patients used to evaluate RA impact on their daily activities. Nevertheless, there are others scores of the functional disability specific of the hand involvement like the Disabilities of the Arm, Shoulder and Hand (DASH) score.^24^ It would have been more appropriate to use this score, since oral hygiene is linked to brushing. This study generated a wealth of information on the oral hygiene status in Moroccan RA patients and related factors. Several limitations should be pointed out. First, because of technics reasons, we did not complete with a clinical examination of the periodontium by a dentist. Second, because the heterogeneous nature of the studied population and the cross-sectional nature of our study, we found low percentages of hyper-tension and dyslipidaemia on the one hand, and on the other hand we did not find any association between regular brushing, RA disease activity, and HAQ. However, our study evaluated oral hygiene in 100 patients and highlighted factors associated with precarious oral hygiene.

We believe that this first work at the national level would have clarified the seriousness of the lack of oral hygiene in this category of patients rarely informed by their rheumatologist, and would have indicate the need for a close relationship between the rheumatologist and the dentist in order to promote appropriate hygiene measures, as well as to treat and prevent potential oral complications.

## CONCLUSION

The present study illustrates the high prevalence of oral hygiene precariousness in RA patients that seems to be more important in men and illiterate patients. It also highlights a poor information given by the rheumatologist on the importance of adequate oral hygiene in the management of RA. It will be interesting to discuss and establish information strategies with rheumatologists for better care of RA patients. It will be interesting also to achieve a close relationship between the rheumatologist and the dentist, in order to treat and prevent potential oral complications. An information and prevention strategy through awareness campaigns, and the establishment of recommendations adapted to our Moroccan socioeconomic environment, seems to be an urgent need.

## ABBREVIATIONS:

ACR: American College of Rheumatology
BP: Blood pressure
Cs DMARDs: Conventional synthetic Disease Modifying Anti Rheumatic Drug
DAS 28: Disease activity score 28
DASH: Disabilities of the Arm, Shoulder and Hand
EULAR: European League Against Rheumatism
FBG: Fasting blood glucose
HAQ: Health Assessment Questionnaire
HDL-C: High-density lipoprotein cholesterol
LDL-C: Low-density lipoprotein cholesterol
RA: Rheumatoid arthritis
RBG: Random blood glucose
SPSS: Statistical Package for the Social Sciences
TC: Total cholesterol
